# Palliative Care Research: Successful Recruitment and Retention Strategies of Patient-Caregiver Dyads

**DOI:** 10.1093/geroni/igaa057.791

**Published:** 2020-12-16

**Authors:** Lissi Hansen, Shirin Hiatt, Karen Lyons

**Affiliations:** 1 Oregon Health & Science University, Portland, Oregon, United States; 2 Boston College, Chestnut Hill, Massachusetts, United States

## Abstract

Research shows that the well-being of patients with serious illness and their family caregivers is significantly associated. Thus, to build the scientific knowledge upon which to establish high quality palliative and end-of-life care practices for these patients and their caregivers, research studies should include successful recruitment and retention strategies that focus on the patient-caregiver dyad. Aims: To review the literature focusing on successful dyadic recruitment and retention strategies and to describe successful recruitment and retention strategies, and attrition in a longitudinal study of end-stage liver disease (ESLD) patient-caregiver dyads. Methods: A five-year prospective longitudinal study of dyads included quantitative and qualitative data collected at 5 time points over 1 year: at baseline, 3, 6, 9, and 12 months. Results: Over a 32-month period 336 dyads were approached and 241 were enrolled. The refusal rate was 27 dyads (8.0%). Over the course of the study, 31 patients or caregivers withdrew for various reasons (too sick, liver transplantation). The attrition due to death of patients was 53 dyads (20.2%). Successful strategies used for recruitment and retention included tailoring to provider preference for referral, accommodating patient preference for data collection method, and having predictable and ongoing contact between a specific study staff and dyads. Conclusions: Less than 10 studies address recruitment and retention strategies most effective in dyadic research in various serious illnesses and clinical settings. Recruitment of ESLD patient-caregiver dyads is challenging. Future longitudinal dyadic studies of serious illnesses and palliative care may benefit from strategies learned from the current ESLD study.

